# Supplementary material for: Seroprevalence of Getah virus in Pigs in Eastern China Determined with a Recombinant E2 Protein-Based Indirect ELISA
Source: Viruses. 2022 Sep 30;14(10):2173. doi: 10.3390/v14102173 (PMC9607375; doi:10.3390/v14102173)
Supplement: Supplementary file 1 [file viruses-14-02173-s001.zip › viruses-1954936-supplementary.pdf]

Supplementary Materials

Table S1. Identification of negative and positive serum for GETV.

Sample	VN	IFA	ELISA	qRT-PCR
GETV-P1*	1:320	+	1.566	+
GETV-P2*	1:640	+	1.632	+
GETV-P3*	1:320	+	1.578	+
GETV-P4*	1:320	+	1.501	+
GETV-P5*	1:640	+	1.788	+
GETV-P6*	1:1280	+	1.945	+
GETV-P7*	1:640	+	1.600	+
GETV-P8*	1:320	+	1.511	+
GETV-P9*	1:320	+	1.536	+
GETV-P10*	1:160	+	1.254	+
GETV-N1*	<1:5	-	0.301	-
GETV-N2*	<1:5	-	0.222	-
GETV-N3*	<1:5	-	0.125	-
GETV-N4*	<1:5	-	0.241	-
GETV-N5*	<1:5	-	0.114	-
GETV-N6*	<1:5	-	0.155	-
GETV-N7*	<1:5	-	0.144	-
GETV-N8*	<1:5	-	0.174	-
GETV-N9*	<1:5	-	0.134	-
GETV-N10*	<1:5	-	0.129	-

"*" means that sera is from naturally infected pig. **"+"**, positive; "-", negative.

ELISA: "X" >0.344, positive; "X"<0.344, negative.

Table S2. Identification of negative and positive serum for JEV, PRRSV, CSFV, PEDV, PRV.

Sample	VN	IFA
JE-P1	1:1280	+
JE-P2	1:640	+
JE-P3	1:320	+
JE-P4	1:320	+
JE-P5	1:160	+
PRRS-P1	1:160	+
PRRS-P2	1:160	+
PRRS-P3	1:320	+
PRRS-P4	1:320	+
PRRS-P5	1:640	+
CSF-P1	1:320	+
CSF-P2	1:640	+
CSF-P3	1:320	+
CSF-P4	1:320	+
CSF-P5	1:640	+
PED-P1	1:320	+
PED-P2	1:640	+
PED-P3	1:640	+
PED-P4	1:640	+
PED-P5	1:640	+
PR-P1	1:512	+
PR-P2	1:512	+
PR-P3	1:512	+
PR-P4	1:512	+
PR-P5	1:512	+

"+", positive; "-", negative.
